# Benchmark evaluation of multi-modal large language models for ophthalmic diagnosis in real world

**DOI:** 10.3389/fmed.2026.1842016

**Published:** 2026-06-22

**Authors:** Shoujun Huang, Junhong Chen, Jiaoman Wang, Ping Zhang, Wending Du, Yuan Hong, Dexing Kong, Wei Lou, Mingying Lai, Weihua Yang

**Affiliations:** 1 College of Mathematical Medicine, Zhejiang Normal University, Jinhua, China; 2 Eye Hospital and School of Ophthalmology and Optometry, Wenzhou Medical University, Wenzhou, Zhejiang, China; 3 Shenzhen Eye Hospital, Shenzhen Eye Medical Center, Southern Medical University, Shenzhen, China

**Keywords:** clinical benchmark dataset, medical image diagnosis, multimodal large language models, ophthalmology, performance evaluation

## Abstract

Multimodal large language models (MLLMs) are increasingly demonstrating substantial potential in the medical domain, particularly in image-intensive specialties such as ophthalmology. Although cutting-edge models like ChatGPT-4o and Qwen-VL 2.5 have shown strong performance on general-domain tasks, real-world clinical benchmarks for rigorously assessing their diagnostic capabilities in specialized medical contexts remain limited. To address this gap, we constructed a carefully curated benchmark dataset comprising 295 pathologically confirmed ophthalmic cases with representative clinical presentations. Using this dataset, we systematically evaluated nine leading MLLMs, including both open-source and proprietary models. The results showed that models such as HAIBU-ReMUD and ChatGPT-4o achieved comparatively strong diagnostic accuracy and consistency, with performance in some settings approaching that of human experts. These findings suggest that current MLLMs are showing encouraging feasibility for real-world clinical applications and provide a basis for further investigation of their integration into ophthalmology practice.

## 1 Introduction

Multi-modal large language models (MLLMs) are increasingly recognized for their potential to revolutionize clinical interpretation of medical images, offering advanced capabilities in diagnostic classification, lesion localization, and cross-modal reasoning (1–5). Recent advancements in MLLMs have enabled these systems to simultaneously process both textual and visual inputs, making them particularly well suited for tasks that require integrating narrative clinical descriptions with high-resolution medical images—such as in radiology, dermatology, and ophthalmology (6–10).

Ophthalmology is a particularly promising field for MLLMs, as many diagnostic decisions rely on the integration of image-based pattern recognition with textual clinical context (11–16). However, rigorous evaluation of MLLMs in this field remains limited, particularly in real-world clinical settings (17, 18). Most prior studies have relied on synthetic benchmarks, offering limited insight into real-world diagnostic performance and clinical applicability (19–24). Although state-of-the-art proprietary models such as ChatGPT-4o (25) and Gemini 2.5 Flash (26) achieve strong performance on general AI benchmarks, their effectiveness in specialized medical fields such as ophthalmology remains insufficiently characterized in real-world diagnostic contexts. Similarly, while medical MLLMs such as HAIBU-ReMUD (27) and HealthGPT (28) have demonstrated strong capabilities in interpreting both medical images and text, their performance on ophthalmology-specific tasks remains incompletely evaluated in clinically grounded settings (29–31). Building on prior work, this study further conducted a systematic and quantitative comparison of the diagnostic performance of general-purpose and medically oriented multimodal large language models (MLLMs) using real-world ophthalmic data and clinically relevant tasks, with the aim of addressing the current lack of evidence in this field.

This study focused on evaluating the performance of these models on real-world ophthalmic cases, with particular attention to their abilities in open-ended clinical question answering, multimodal information integration, and natural language reasoning. To ensure consistency and comparability, all included models were interactive MLLMs capable of prompt-based reasoning and suitable for evaluation within a unified open-ended case-based benchmarking framework; additionally, they were not primarily developed or optimized for ophthalmology as a single specialty domain. Accordingly, we included models with capabilities in open-ended visual question answering, cross-modal clinical reasoning, and natural language diagnostic generation, encompassing both proprietary and open-source systems as well as general-purpose and medically oriented MLLMs. In contrast, ophthalmology-specific models that had been deeply adapted or optimized for specialty ophthalmic tasks, as well as ophthalmic vision foundation models primarily intended for representation learning or closed-set classification, were not included in the present evaluation.

To evaluate the specific performance levels of each model, we curated a comprehensive benchmark dataset of ophthalmic diagnostic cases from peer-reviewed journals, integrating expert-written clinical narratives with authentic medical images. Specifically, the dataset includes 151 cases with pathologically confirmed typical clinical presentations from 2023 and 144 cases from 2024, all drawn from leading ophthalmology journals such as *Ophthalmology* and *JAMA Ophthalmology*. Final diagnoses were determined through a standardized assessment protocol conducted by two attending ophthalmologists, with the goal of precisely evaluating performance differences among the models in diagnosing ophthalmic cases.

## 2 Dataset and method

### 2.1 VQA challenge dataset

Diagnostic cases for this study were sourced from two internationally recognized ophthalmology journals: the “Pictures & Perspectives” section of Ophthalmology and the “Clinical Challenge” section of JAMA Ophthalmology. These sections are editorially curated to present diagnostically informative, educationally valuable, and often clinically challenging ophthalmic cases. Each case includes three key components: high-quality clinical images, an English-language narrative describing patient presentations, and reference diagnoses provided by expert ophthalmologists, thereby forming a complete multimodal diagnostic evidence chain.

To ensure broad ophthalmic coverage, cases were initially categorized according to pathological type, including corneal disorders, retinal diseases, glaucoma, eyelid and lacrimal disorders, uveitis, ocular infections, and tumors. Three exclusion criteria were applied: (1) insufficient image quality for diagnostic interpretation; (2) lack of essential diagnostic information in textual descriptions; and (3) diagnoses relying primarily on laboratory findings rather than clinical and imaging evidence.

The final dataset contains 295 ophthalmic cases spanning multiple subspecialties, including optic nerve and glaucoma-related disorders (ONGD, 10 cases), uveal diseases (UD, 19 cases), retinal diseases (RD, 74 cases), ocular surface diseases (OSD, 57 cases), anterior segment disorders including lens diseases (ASDL, 19 cases), ocular trauma (OT, 7 cases), intraocular foreign bodies (IOFB, 2 cases), orbital disorders (OD, 15 cases), lacrimal system diseases (LSD, 6 cases), adverse drug reactions (ADR, 3 cases), and other rare ophthalmic conditions (OROC, 83 cases). For brevity, disease categories were abbreviated in the subsequent Table 1.

**TABLE 1:** Average performance scores of different models across ophthalmic disease categories.

Categories	HB	GPT4	Gemi	Gem4	Gem12	HeG	Int	Med	Qw
ONGD	3.75	4.05	3.15	2.70	3.00	**4.20**	3.00	3.35	3.35
UD	4.21	**4.29**	3.60	3.79	3.29	3.39	3.08	3.76	3.45
RD	**4.12**	4.01	3.71	3.56	3.50	3.30	3.16	3.22	3.43
OSD	3.90	**4.02**	3.88	3.47	3.68	3.53	3.18	3.54	3.74
ASDL	**4.05**	**4.05**	3.66	3.55	3.45	3.47	2.92	3.87	3.37
OT	**4.64**	3.93	3.14	3.71	3.79	3.86	3.64	3.07	4.00
IOFB	4.75	4.50	3.00	4.25	**5.00**	3.75	3.75	2.25	3.75
OD	**3.87**	3.80	3.83	3.30	3.43	3.20	3.47	3.57	2.60
LSD	3.83	**4.25**	3.75	3.75	3.33	2.75	3.25	2.92	4.00
ADR	**4.33**	3.50	3.67	3.17	3.00	3.33	2.67	2.67	3.33
OROC	**4.11**	4.01	3.85	3.64	3.52	3.24	3.62	3.43	3.51

Disease categories and model names are abbreviated. Bold values indicate the highest score in each category, and underlined values indicate the second-highest score.

Note that the source materials were derived from leading ophthalmology journals, and the dataset was intentionally constructed to include diagnostically challenging, atypical, or rare ophthalmic cases, with the aim of establishing a benchmark of substantial diagnostic complexity for evaluating the multimodal reasoning ability, diagnostic robustness, and generalization capability of MLLMs across diverse ophthalmic scenarios.

For data organization, each original document was systematically processed to extract structured elements. Unique identifiers and diagnostic annotations were recorded in tabular form. Relevant visual content was isolated and stored as individual image files, while associated textual descriptions were transcribed into separate textual records to preserve alignment between visual and linguistic information. The corresponding image paths, textual descriptions, and reference diagnoses were subsequently integrated into a unified benchmark dataset for model evaluation.

### 2.2 Question-answer pair design and model access

In the multimodal analysis framework, each paired input consisted of an ophthalmic image and its corresponding clinical text, which were jointly provided to the MLLMs as an integrated image–text case. To ensure consistency across models and facilitate comparative evaluation, we applied a standardized prompting strategy to all models using the following instruction: “ < case report > . Based on the provided image and clinical information, please make an ophthalmic diagnosis for this patient. Output the diagnostic conclusions only.” All models were evaluated in a zero-shot setting without ophthalmology-specific adaptation, additional domain-specific fine-tuning, or task-specific optimization.

Model selection followed predefined inclusion criteria. All included models were interactive multimodal large language models (MLLMs) capable of prompt-based reasoning and suitable for a unified open-ended case-based benchmarking framework. These models were not primarily developed or optimized for ophthalmology as a single specialty domain. Therefore, this benchmark focuses on contemporary conversational MLLMs, including general-purpose and medically oriented systems. Ophthalmology-specific vision foundation models were not included in this evaluation.

To establish a benchmark for current multimodal AI capabilities in ophthalmic diagnosis, we selected a diverse and representative set of MLLMs with the ability to process both visual and textual clinical information. The models evaluated included ChatGPT-4o (GPT4; (25)), Gemini 2.5 Flash (Gemi; (26)), HAIBU-ReMUD (HB; (27)), HealthGPT (HeG; (28)), InternVL 3-8B (Int; (32)), Qwen-VL 2.5-7B (Qw; (33)), MedGemma (Med; (34)), Gemma 3-4B (Gem4; (35)) and Gemma 3-12B (Gem12; (35)).

For open-source models, including Qwen-VL, InternVL, Gemma, and MedGemma, local deployment was performed on NVIDIA RTX 4090 GPUs for batched inference. To improve reproducibility and minimize stochastic variation in model outputs, the decoding parameters were fixed at a temperature of 0.01 and top_p of 1. Closed-source models, including ChatGPT-4o and Gemini 2.5 Flash, were accessed through their official application programming interfaces APIs using the same standardized input format and prompting strategy.

### 2.3 Likert five-point scale scoring mechanism

All image data used in this study were sourced from published journal articles and accompanied by authoritative reference diagnoses. The original cases published in the journals already contained expert-confirmed standard diagnostic answers, which were treated as the diagnostic ground truth in this study. The ophthalmologists therefore evaluated model-generated responses based on their degree of agreement with these reference diagnoses rather than through unrestricted subjective judgment. The question–answer pairs were designed by professional ophthalmologists to accurately represent the original cases while allowing flexibility in model responses. Because the model outputs were in free-text form, a simple semantic or label matching approach could not accurately assess clinical understanding and diagnostic quality. As a result, expert manual evaluation was employed.

The model outputs were assessed by two attending ophthalmologists, each with over 20 years of clinical experience. Their evaluation covered both the accuracy of the diagnostic labels and the overall medical quality and clinical relevance of the generated text.

The two physicians independently scored the diagnostic accuracy of the model’s outputs using a five-point Likert scale. All scoring was performed with reference to the official diagnoses provided in the original journal publications. To objectively define scoring discrepancies, a pre-defined threshold for re-evaluation was established: if the initial ratings of the two physicians differed by ≥ 2 points, the case was considered to have a significant discrepancy. Before the formal evaluation, both assessors jointly reviewed five randomly selected cases and consulted established clinical guidelines from UpToDate and PubMed to standardize their scoring criteria and assessment approach.

After this calibration phase, one assessor conducted the primary evaluation, while the second independently reviewed all scores. For cases with significant discrepancies in the initial ratings, the two ophthalmologists re-examined the model outputs together and reached a consensus score through discussion. The results revealed that cases requiring this re-evaluation procedure accounted for 4.7% of the total. After re-evaluation, the final score difference for all cases was ≤1 point, thereby ensuring overall consistency of the scoring results.

### 2.4 Statistical analyses

Statistical analyses were performed using the R software environment. Inter-rater agreement and potential bias were assessed using Bland–Altman plots (36), with consistency between raters further visualized through scatter plots and heatmaps. The distribution of model scores was illustrated using density curves and boxplots, while variance in model predictions was compared using Friedman tests to determine whether differences among models were statistically significant.

## 3 Results and discussion

### 3.1 Statistical visualization analysis

A total of 295 ophthalmic clinical cases were included, covering a broad range of disease types and reflecting the diversity of disease distribution in real-world clinical practice. The results revealed a clear performance stratification among the evaluated models. HAIBU-ReMUD and ChatGPT-4o achieved the highest overall scores and demonstrated relatively strong stability, with outputs in most cases closely approximating expert-level performance. The Gemma 3 series (4B/12B) and Gemini models showed intermediate performance, although some variability across disease categories was observed, suggesting limited consistency in cross-task generalization. Qwen-VL 2.5 and HealthGPT also fell within the mid-performing group but remained below the top-performing models. In contrast, InternVL 3-8B and MedGemma obtained comparatively lower scores overall, indicating substantial room for improvement in model architecture, training strategy, or domain-specific adaptation.

**FIGURE 1:**
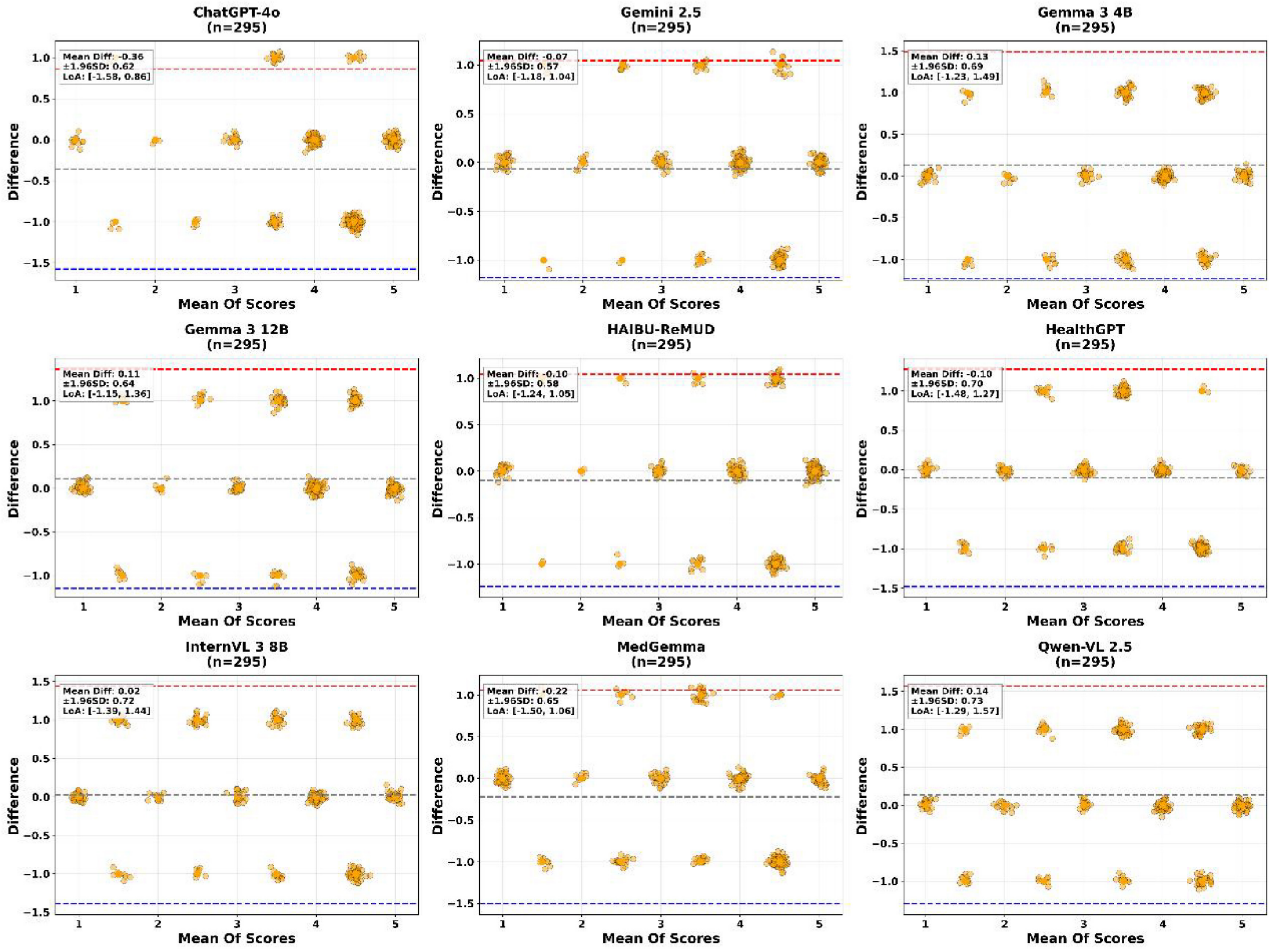
Bland-Altman plots comparing scores between two physicians’ assessments.

**FIGURE 2:**
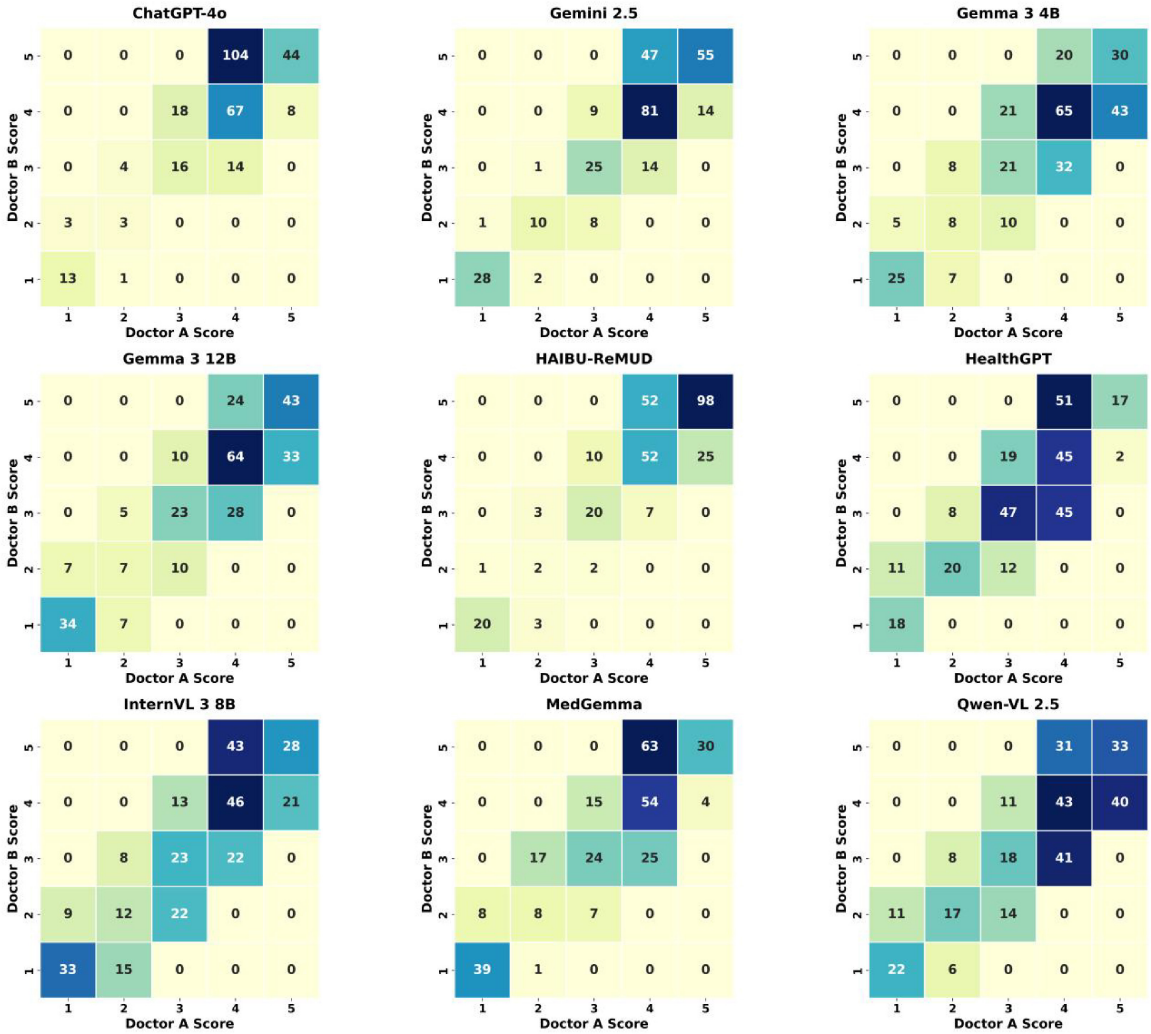
Nine heatmaps respectively show the score distributions of different models.

**FIGURE 3:**
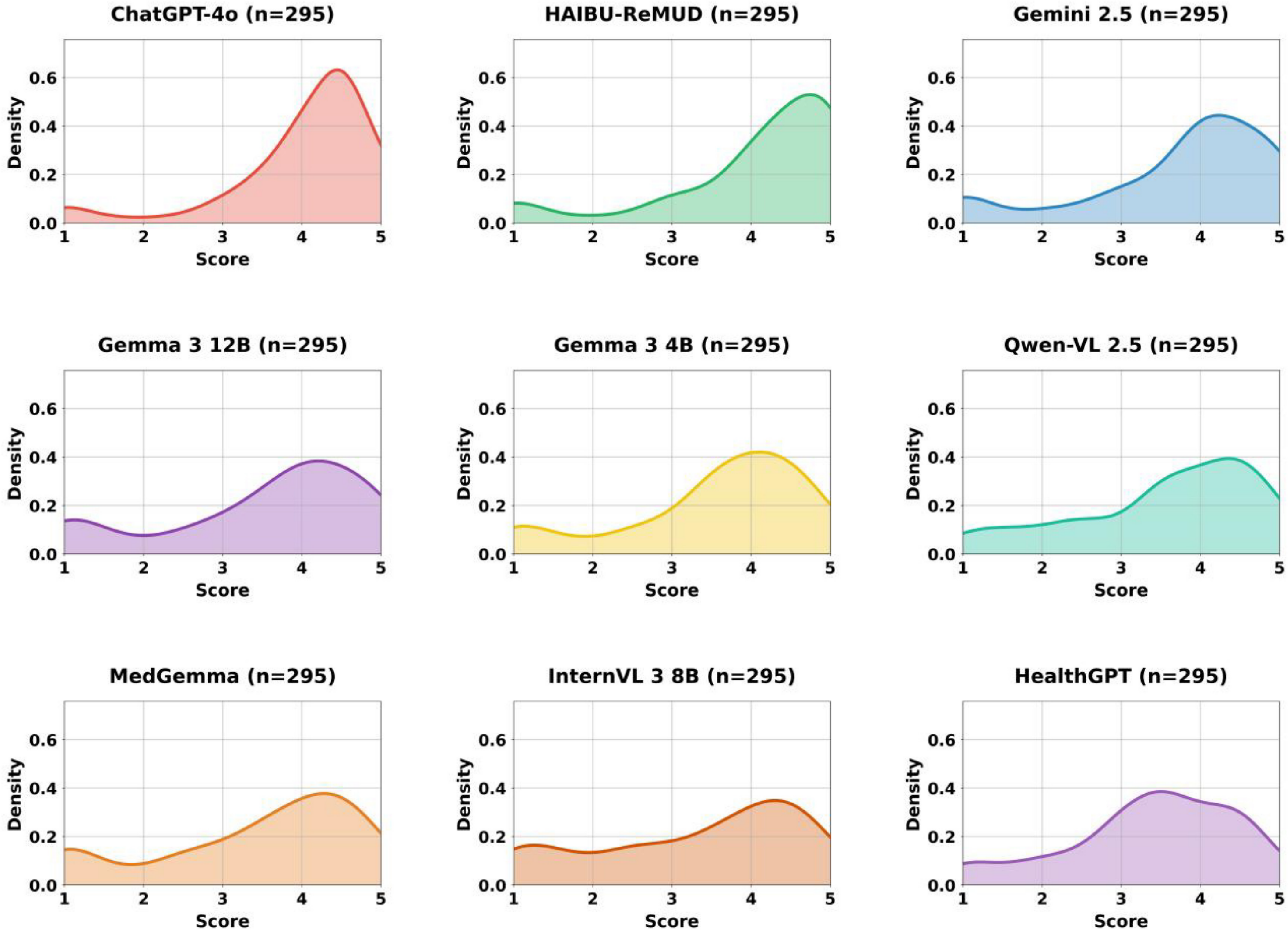
The nine density plots respectively show the score distributions of different models.

**FIGURE 4:**
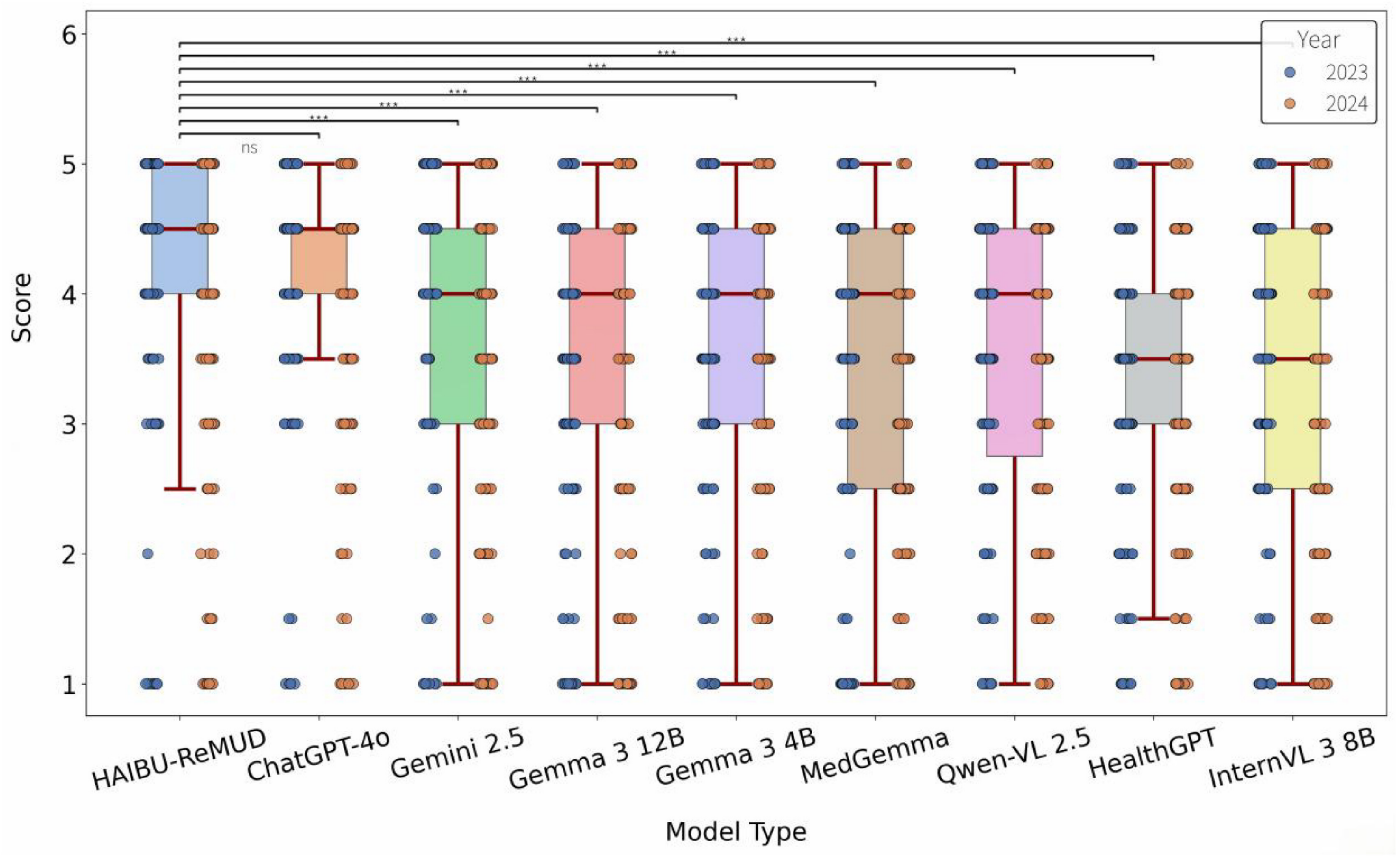
Box-and-whisker plots that depict the score distributions stratified by different model types.

To further validate these findings, key statistical visualizations are provided: Figure 1 presents Bland-Altman plots to assess inter-rater agreement and potential bias; Figures 2, 3 clearly illustrate the score distribution characteristics across the nine models; and Figure 4 utilizes box plots (37) to corroborate the density analysis results, revealing statistically significant differences among the models. In addition, Table 1 summarizes the average performance scores of the different models across ophthalmic disease categories, thereby providing a category-level comparison that further complements the analysis of performance differences among models.

#### 3.1.1 Bland-Altman plots analysis

Each Bland-Altman plot illustrates the relationship between the mean scores from both physicians (*X*-axis) and the difference between their scores (*Y*-axis). The agreement is quantified using the mean difference (bias) and 95% limits of agreement (LoA). The LoA are calculated as the mean difference ± 1.96 times the standard deviation of the differences, defining the range within which 95% of the score differences between the two raters are expected to lie. A bias closer to zero combined with narrower LoA indicates stronger consensus and lower variability between the raters. Each orange dot represents an individual case. Overall, the analysis reveals varying degrees of scoring consistency among the models.

In Figure 1, Gemini 2.5 exhibited the most consistent performance, with a small bias (−0.07) and the narrowest limits of agreement (LoA) (−1.18 to +1.04), indicating minimal systematic bias and low random variability. HAIBU-ReMUD (−0.10) and HealthGPT (−0.10) also showed strong agreement, with relatively tight LoA.

Notably, InternVL 3-8B achieved the smallest bias (+0.02), suggesting nearly no systematic difference between raters. However, its LoA (−1.39 to +1.44) was among the widest, indicating substantial random variability despite good average agreement.

In contrast, Qwen-VL 2.5 (+0.14) exhibited the largest positive bias, while ChatGPT-4o (−0.36) and MedGemma (−0.22) showed significant negative biases. Gemma 3 12B (−1.13 to +1.36) and Qwen-VL 2.5 (−1.29 to +1.57) displayed the widest LoA and most dispersed points, reflecting the greatest variability and poorest consistency.

#### 3.1.2 Score distribution analysis

In each 5 × 5 heatmap, the *x*-axis and *y*-axis represent the rating levels (1–5) given by two physicians, while the color intensity indicates the frequency of each score combination, reflecting the consistency pattern between expert evaluations.

In each density plot, the *x*-axis represents the average physician rating for each model output (ranging from 1.0 to 5.0), reflecting overall model performance, while the *y*-axis denotes the probability density of the scores. Each subplot shows the score distribution of a specific model using a density curve, where the peak indicates the most frequently occurring score range.

Figure 2 illustrates the consistency pattern between expert evaluations. To further highlight the central tendency of model output scores, Figure 3 presents the corresponding density distribution plots, providing an intuitive comparison of score differences and performance variability across the models.

Specifically, the curve for HAIBU-ReMUD shows a distinct right skew, with its main peak sharply concentrated in the 4.5–5.0 range. This indicates a high score density, low dispersion, and overall stability, confirming its reliability as a benchmark. The distributions for ChatGPT-4o and Gemini 2.5 closely resemble HAIBU-ReMUD’s, with significantly increased density in the high-score region (above 4.0), reflecting strong output capability and consistency, which solidly places them in the top tier. The Gemma 3 series (4B/12B) also exhibits a right-skewed distribution, indicating generally good performance, though their distributions are broader with more pronounced tails, suggesting slightly lower output stability. MedGemma shows a more complex shape, with a subtle bimodal trend, which may indicate unbalanced performance and fluctuating adaptability across different task scenarios.

In contrast, the curves of InternVL 3-8B, HealthGPT, and Qwen-VL 2.5 are notably shifted leftward to the mid-to-low score range (2.0–3.5). These distributions are characterized by low, flat density, significant fluctuations, and long tails, indicating insufficient output quality and difficulty in consistently generating high-quality responses.

#### 3.1.3 Box-and-Whisker plots analysis

In Figure 4, the *x*-axis represents “Model Type,” corresponding to each model included in the evaluation; the *y*-axis represents the “Score” ranging from 1.0 to 5.0, serving as the model performance metric. Each box plot displays the interquartile range (IQR) of the scores, with the horizontal line inside the box marking the median, and the whiskers extending to the minimum and maximum values within 1.5 times the IQR. Data points from 2023 are indicated in blue, and those from 2024 in orange, forming discrete distributions. Overlapping occurs in the plot due to the concentrated distribution of some data points. Statistical significance between models was assessed using the Friedman test. This visualization enables comparative analysis of the central tendency, variability, and potential outliers in the score distributions across the different models.

As shown in Figure 4, HAIBU-ReMUD demonstrates near-perfect median scores, with an exceptionally compact interquartile range (IQR) and very few outliers, underscoring its remarkable scoring consistency. The Friedman test revealed no statistically significant difference between HAIBU-ReMUD and ChatGPT-4o (*p* > 0.05), while highly significant differences (*p* < 0.001) were observed compared to all other models, solidifying HAIBU-ReMUD as the only benchmark-level model consistently maintaining scores above 4.5. The box plots for ChatGPT-4o, Gemini 2.5, and Gemma 3 4B appear tall yet compact, with minimal outliers. Although both Gemini 2.5 and Gemma 3 4B show statistically significant differences from HAIBU-ReMUD (*p* < 0.001), all three models demonstrate high scoring reliability, as evidenced by their concentrated upper quartiles and limited dispersion of outliers.

Gemma 3 12B and MedGemma show median scores close to 4.0 and also differ statistically from HAIBU-ReMUD (*p* < 0.001). Notably, these two models display larger IQRs, indicating greater volatility in output quality. Among them, MedGemma exhibits particularly prominent score dispersion, with numerous outliers at both high and low score ranges. This suggests that while it occasionally generates high-quality responses, its performance stability is significantly lacking.

#### 3.1.4 Model performance across ophthalmic disease categories

Table 1 presents the differences in mean scores of various models across 11 ophthalmic disease categories. Overall, HAIBU-ReMUD and ChatGPT-4o demonstrated the most outstanding performance, achieving relatively high scores in most categories, which indicates stronger cross-disease generalization ability and more stable task adaptability. In contrast, the performance of Gemini 2.5 Flash, the Gemma 3 series, InternVL 3-8B, MedGemma, and Qwen-VL 2.5-7B showed greater variability, with more pronounced differences across categories, suggesting that their capabilities remain uneven in specific ophthalmic subspecialty scenarios.

From the category-wise distribution, most models achieved relatively higher scores in tasks related to ocular trauma (OT), intraocular foreign bodies (IOFB), and other rare ophthalmic conditions (OROC), whereas their performance was comparatively weaker in adverse drug reactions (ADR), orbital disorders (OD), and some optic nerve and glaucoma-related disorders (ONGD). These findings suggest that different disease categories impose varying demands on the models’ clinical reasoning, differential diagnosis, and fine-grained visual understanding abilities.

### 3.2 Discussion

Based on 295 real-world ophthalmic clinical cases, this study establishes a systematic benchmarking framework for multimodal large language models (MLLMs) and reveals pronounced performance stratification among the nine evaluated models. Notably, HAIBU-ReMUD and ChatGPT-4o demonstrated superior stability and tightly clustered performance distributions, suggesting more reliable and human-like reasoning behavior in complex clinical decision-making scenarios (38, 39, 40).

These findings suggest that the consistency of clinical reasoning is not determined solely by model scale or parameter count, but is instead largely influenced by the quality of cross-modal alignment and the extent to which domain-specific medical knowledge is incorporated. For example, the strong zero-shot performance of HAIBU-ReMUD may be related to the inclusion of relatively rich medical textbook content in its training corpus, which may also contain ophthalmic knowledge. This knowledge-supported training paradigm may enable the model to generalize more effectively to previously unseen cases without task-specific fine-tuning. This observation further underscores the important value of domain-informed learning in real-world medical applications.

Compared with previous ophthalmic MLLM studies (41, 42, 43), the main contribution of this work lies in further developing and refining, on the basis of existing related research, a quantitative benchmarking framework that is oriented toward real-world settings, integrates multimodal information, and is grounded in clinical practice. This benchmark incorporates multiple common ophthalmic imaging modalities and supports systematic comparison across mainstream MLLMs. Such a design more closely reflects the complexity of real clinical practice and also provides a foundation for further analysis of model robustness, interpretability, and error patterns. In addition, our integrated evaluation framework combines consistency metrics with visualization-based analytical methods, forming a clinically oriented validation pipeline with good reproducibility and offering a valuable methodological reference for future research in multimodal medical artificial intelligence.

Despite the promising performance of top-tier models, substantial challenges remain before clinical deployment. We observed a subset of model responses that were medically inaccurate despite being linguistically fluent and superficially plausible. A brief *post hoc* qualitative review suggested that these failures reflected heterogeneous error patterns rather than a single mechanism, including omission or underestimation of decisive clinical findings, insufficient integration of multimodal evidence, and premature diagnostic closure. Representative examples included a case in which the model underrecognized a decisive dynamic clinical feature—a conjunctival-corneal membrane freely mobile over the ocular surface—and consequently generated a plausible but insufficiently supported interpretation of an ocular surface lesion, as well as a case in which a hemi-macular OCT abnormality was discussed only in terms of etiologic possibilities without recognition of the lesion-specific diagnosis. Such issues may be further amplified by overconfident language generation, producing outputs that appear authoritative despite being factually incorrect and thereby posing potential safety risks in clinical decision-support settings.

Several limitations of this study should also be acknowledged. First, although expert-based human evaluation provides clinically meaningful judgment and remains important for assessing complex multimodal reasoning, it also has inherent limitations in scalability and long-term reproducibility. Manual assessment is labor-intensive, time-consuming, and may be influenced by inter-rater variability, making it difficult to sustain for large-scale or continuously updated benchmarking. Future work should therefore explore more standardized evaluation frameworks that combine structured diagnostic taxonomies with semi-automated assessment strategies, thereby improving efficiency, consistency, and reproducibility while preserving clinical relevance.

Second, this study aims to evaluate the performance of general-purpose and medically oriented multimodal large language models (MLLMs) in real-world ophthalmic cases, with a particular focus on their transferability and practical applicability in specialist ophthalmic settings. Highly ophthalmology-specialized models, such as VisionUnite (44) and EyeCareGPT (45), were not included in this study. This design enables a more focused evaluation of the baseline capabilities of general-purpose and medically oriented MLLMs in real-world ophthalmic tasks; however, it also limits direct comparison with domain-specialized models, thereby constituting a scope limitation of the study. Accordingly, the exclusion of ophthalmology-specialized models represents a limitation of this work and further highlights the need for future benchmark studies to systematically compare ophthalmology-specific models with general-purpose and medically oriented MLLMs. Such studies are necessary to more comprehensively characterize the complementary strengths of broad multimodal generalization and domain-specific expertise (46).

Looking ahead, multimodal medical AI should move beyond pattern recognition and text generation toward the development of interpretable pathology–symptom–decision reasoning chains, enabling truly clinically explainable AI (CE-AI). Future systems should also incorporate human-in-the-loop validation, continuous clinical learning, and regulatory-grade auditability to ensure long-term accuracy, safety, and ethical compliance. Only through such advances can multimodal medical intelligence evolve from laboratory research into a trustworthy real-world clinical decision-support tool (47, 48).

## 4 Conclusion

In an evaluation of 295 complex ophthalmology clinical cases, HAIBU-ReMUD and ChatGPT-4o secured top-tier positions with excellent stability and high scores, while the remaining models generally exhibited lower scores and insufficient stability. However, even for the best-performing models, reliable integration into mainstream clinical practice will require addressing three core challenges: effectively controlling model hallucinations, achieving seamless integration and alignment of multimodal data, and establishing safety verification systems that meet clinical standards.

## Data Availability

The original contributions presented in this study are included in this article/supplementary material, further inquiries can be directed to the corresponding authors.
